# Supplementary material for: Complex‐centric proteome profiling by SEC‐SWATH‐MS
Source: Mol Syst Biol. 2019 Jan 14;15(1):e8438. doi: 10.15252/msb.20188438 (PMC6346213; doi:10.15252/msb.20188438)
Supplement: Supplementary file 8 — Dataset EV7 [file MSB-15-e8438-s008.zip › feature_plots_string/P40227.pdf]

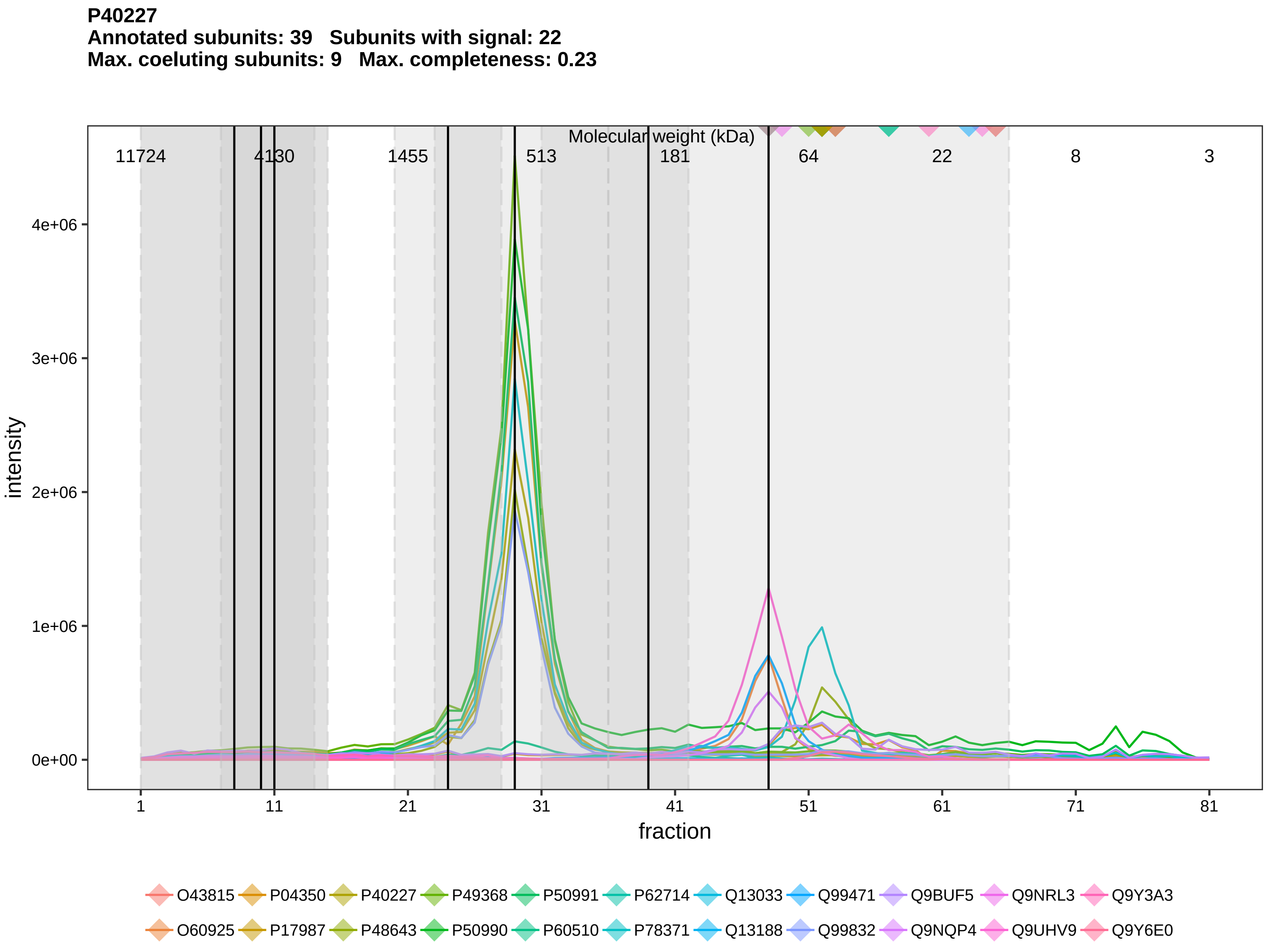
P40227
Annotated subunits: 39   Subunits with signal: 22
Max. coeluting subunits: 9   Max. completeness: 0.23
Molecular weight (kDa)
11724
4130
1455
513
181
64
22
8
3
intensity
4e+06
3e+06
2e+06
1e+06
0e+00
fraction
1
11
21
31
41
51
61
71
81
O43815
P04350
P40227
P49368
P50991
P62714
Q13033
Q99471
Q9BUF5
Q9NRL3
Q9Y3A3
O60925
P17987
P48643
P50990
P60510
P78371
Q13188
Q99832
Q9NQP4
Q9UHV9
Q9Y6E0
